# Supplementary material for: Human learning is an understudied but promising lever for boosting human–AI synergy
Source: Proc Natl Acad Sci U S A. 2026 Sep 21;123(39):e2536100123. doi: 10.1073/pnas.2536100123 (PMC13624608; doi:10.1073/pnas.2536100123)
Supplement: Supplementary file 1 [file pnas.2536100123.sapp.pdf]

Supporting Information for

Human learning is an understudied but promising lever for boosting human–AI synergy

Julian Berger, Jason W. Burton, Ralph Hertwig, Thomas Kosch, Ralf H. J. M. Kurvers, Benito Kurzenberger, Christopher Lazik, Linda Onnasch, Tobias Rieger, Anna I. Thoma, Dirk U. Wulff, Stefan M. Herzog

Julian Berger & Stefan Herzog.
E-mail: berger@mpib-berlin.mpg.de & herzog@mpib-berlin.mpg.de

This PDF file includes:

Supporting text
SI References

Supporting Information Text

Methods.

Data. Vaccaro et al. (1) provided only human-only, AI-only and human–AI performance averaged across all trials of a study condition. While it would be ideal to test our research questions about human learning as a function of how many trials a participant has already engaged with, only three of the 10 studies with feedback reported results as a function of trial number. Thus, the data aggregated by Vaccaro et al. does not allow for such detailed individual-level analysis of potential learning effects.

Moreover, only some studies in Vaccaro et al. openly share their data, and even fewer provide the data needed to reconstruct how participants chose across trials (e.g., (2) Individual participant-level meta analysis remains our overall goal (3), but it requires a new systematic review that treats open data and clear trial sequences as inclusion criteria. In the meantime, we present the within-study analyses of three published datasets in our OSF repository available at https://osf.io/mqw4y and show that learning is both detectable and that accounting for it paints a less pessimistic picture of how humans and machines perform together.

Study design features. Across the 370 effect sizes in (1), we coded the following additional moderators during our systematic review.

- For the presence or absence of feedback during the main task (`feedback_main`), 272 effect sizes had no feedback while 98 had feedback.
- Among cases where feedback was provided (`feedback_main_n`), 94 effect sizes involved feedback on every trial and 4 involved feedback every 30 trials.
- Practice trials were available (`practice_available`) in 155 effect sizes and unavailable in 215.
- The number of trials participants completed in the main tasks (`number_responses`) had a median count of 30 (IQR = 15 to 100), and a mean count of 70.8 (SD = 80.12).
- All of the 370 effect sizes involved single-task scenarios as opposed to participants engaging in multiple tasks at once (`multitask`) and were conducted in artificial laboratory or online settings (`setting`) as opposed to natural or field experiments.
- Regarding the number of experimental sessions (`sessions`), 332 effect sizes came from studies with one single session while 38 came from studies that invited participants into multiple sessions.
- With regard to performance incentives (`incentive`), 88 effect sizes involved no incentive, 141 involved performance-based incentives, and 141 provided no information about incentives.
- The level of automation (`automation`) varied: 283 effect sizes involved systems providing one specific solution, 85 involved systems providing multiple solutions, and 2 involved systems showing and implementing solutions unless vetoed by the human participant.
- Finally, the sequence of human–AI decision making (`sequence`) was concurrent in 236 effect sizes (i.e., participants deciding while observing system output), sequential in 114 (i.e., participants deciding independently first before being able to revise with AI), on-demand in 8 (i.e., participants being able to request assistance), time-delayed in 8 (i.e., participants needing to wait for some time before seeing system output), and involved the system offering advice when deemed necessary as judged by the AI in 4 instances.

Studies with feedback. Ten studies provided performance feedback in the main task, contributing 98 effect sizes across 14 different tasks. Most tasks involved classification or prediction decisions: (4) had participants predict recidivism with and without AI explanations (total number of effect sizes contributed to meta-analyses, n = 12); (5) examined classifying beer reviews, Amazon reviews, and answering LSAT questions with and without AI explanations (each n = 12); (6) studied baggage x-ray screening without AI explanations (n = 3); (7) examined basketball game predictions without explanations (n = 6); (8) studied image labeling with and without AI explanations (n = 5); (9) examined sorting recyclables without explanations (= 1); (10) studied image classification without explanations (n = 48); (11) examined leaf and liver image classification with and without explanations (each n = 6); and (12) studied shape classifications without explanations (n = 8). Only one study involved a creation task: (13) examined code translation without AI explanations (n = 6).

Robust Bayesian Model Averaging. We used a Robust Bayesian Model Averaging (RoBMA; Rpackage version 3.5) approach for our meta-regressions evaluating moderation effects (14, 15). This approach runs many models, systematically varying assumptions (e.g., absence versus presence of main or moderator effects, absence versus presence of publication bias) and then calculates a posterior effect-size estimate based on the average of all models, weighted by how well the models account for the data. The resulting meta-regression models quantify the evidence for moderation using Bayes factors ($BF_{inclusion}$) and make it possible to calculate the conditional marginal posterior effect-size estimate for different moderator levels (i.e., feedback versus no feedback).

We used R 4.4.2. for all analyses. Our RoBMA models have the same three-level hierarchical model structure originally used by Vaccaro et al. (1), nesting effect sizes within experiment and experiments within study. We relied on default priors as specified by the RoBMA authors (15). Detailed model summaries and fitted model objects are provided in the project repository available at https://osf.io/mqw4y. See Bartoš et al. (14, 15) and https://fbartos.github.io/RoBMA/ for guidance on how to interpret those outputs.

We fit all models using the spike-and-slab algorithm. Models were run for at least 45,000 samples after 15,000 burn-in and 10,000 adaptive samples. In case models did not converge, burn-in and adaptive samples were increased up to 25,000 and 20,000 respectively. Convergence was checked using the Gelman–Rubin criterion $\hat{R} < 1.05$, relying on the automatic convergence checks implemented in RoBMA.

To calculate the contrast between effect-size estimates of Hedge's g for studies with versus without feedback, we extracted the conditional marginal posterior distributions from the RoBMA models and summarized the resulting posterior distribution of the difference in effect-size estimates using `tidybayes::median_qi()` (16). We also describe the posterior difference using the probability of direction, using `bayestestR::p_direction()` (17) to quantify how much of the posterior distribution of the difference is greater than 0.

References

1. M Vaccaro, A Almaatouq, T Malone, When combinations of humans and AI are useful: A systematic review and meta-analysis. *Nat. Hum. Behav.* **8**, 2293–2303 (2024).
2. F Poursabzi-Sangdeh, DG Goldstein, JM Hofman, JW Wortman Vaughan, H Wallach, Manipulating and measuring model interpretability in *Proceedings of the 2021 CHI Conference on Human Factors in Computing Systems.* (ACM, Yokohama Japan), pp. 1–52 (2021).
3. H Cooper, EA Patall, The relative benefits of meta-analysis conducted with individual participant data versus aggregated data. *Psychol. Methods* **14**, 165 (2009).
4. Y Alufaisan, LR Marusich, JZ Bakdash, Y Zhou, M Kantarcioglu, Does explainable artificial intelligence improve human decision-making? *Proc. AAAI Conf. on Artif. Intell.* **35**, 6618–6626 (2021) Number: 8.
5. G Bansal, et al., Does the whole exceed its parts? The effect of ai explanations on complementary team performance in *Proceedings of the 2021 CHI Conference on Human Factors in Computing Systems.* (ACM, Yokohama Japan), pp. 1–16 (2021).
6. MM Boskemper, ML Bartlett, JS McCarley, Measuring the efficiency of automation-aided performance in a simulated baggage screening task. *Hum. Factors: The J. Hum. Factors Ergonomics Soc.* **64**, 945–961 (2022).
7. H Elder, C Canfield, DB Shank, T Rieger, C Hines, Knowing when to pass: the effect of ai reliability in risky decision contexts. *Hum. Factors: The J. Hum. Factors Ergonomics Soc.* **66**, 348–362 (2024).
8. G He, L Kuiper, U Gadiraju, Knowing About Knowing: An Illusion of Human Competence Can Hinder Appropriate Reliance on AI Systems in *Proceedings of the 2023 CHI Conference on Human Factors in Computing Systems.* (ACM, Hamburg Germany), pp. 1–18 (2023).
9. H Kyriacou, A Ramakrishnan, J Whitehill, Learning to work in a materials recovery facility: can humans and machines learn from each other? in *LAK21: 11th International Learning Analytics and Knowledge Conference.* (ACM, Irvine CA USA), pp. 456–461 (2021).
10. H Tejeda, A Kumar, P Smyth, M Steyvers, AI-assisted decision-making: a cognitive modeling approach to infer latent reliance strategies. *Comput. Brain & Behav.* **5**, 491–508 (2022).
11. F Yang, Z Huang, J Scholtz, DL Arendt, How do visual explanations foster end users' appropriate trust in machine learning? in *Proceedings of the 25th International Conference on Intelligent User Interfaces.* (ACM, Cagliari Italy), pp. 189–201 (2020).
12. Q Zhang, ML Lee, S Carter, You Complete Me: Human-AI Teams and Complementary Expertise in *Proceedings of the 2022 CHI Conference on Human Factors in Computing Systems*, CHI '22. (Association for Computing Machinery, New York, NY, USA), pp. 1–28 (2022).
13. JD Weisz, et al., Better together? an evaluation of ai-supported code Translation in *27th International Conference on Intelligent User Interfaces.* (ACM, Helsinki Finland), pp. 369–391 (2022).
14. F Bartoš, M Maier, EJ Wagenmakers, Robust Bayesian multilevel meta-analysis: Adjusting for publication bias in the presence of dependent effect sizes (2025).
15. F Bartoš, M Maier, TD Stanley, EJ Wagenmakers, Robust Bayesian meta-regression: Model-averaged moderation analysis in the presence of publication bias. *Psychol. Methods* (2025).
16. M Kay, *tidybayes: Tidy data and geoms for Bayesian models.* (2024).
17. D Makowski, MS Ben-Shachar, D Lüdecke, bayestestR: Describing effects and their uncertainty, existence and significance within the Bayesian framework. *J. Open Source Softw.* **4**, 1541 (2019).
